# A Comprehensive Review on Solid Lipid Nanoparticles as a Carrier for Oral Absorption of Phyto-Bioactives

**DOI:** 10.7759/cureus.68339

**Published:** 2024-08-31

**Authors:** Gokul V, Pavithra Kothapalli, Manimaran Vasanthan

**Affiliations:** 1 Department of Pharmaceutics, SRM College of Pharmacy, SRM Institute of Science and Technology, Kattankulathur, IND

**Keywords:** bioavailability, encapsulation, targeted delivery, solid lipid nanoparticles, bioactives

## Abstract

Phyto-bioactive (PB) compounds are naturally occurring substances derived from plants that offer significant health benefits ranging from antioxidant and anti-inflammatory activities to potential cancer-fighting properties. However, their widespread application is limited by several inherent limitations, such as low bioavailability, poor biostability, limited aqueous solubility, and no site-specific target. Additionally, the necessity for high concentrations of effective PBs doses further restricts their use. Encapsulating PBs in suitable nanocarriers, particularly solid lipid nanoparticles (SLNs), can enhance their stability in biological environments, improve water solubility, enable controlled release, and allow for targeted delivery. This innovative approach increases bioavailability, reduces toxicity, and potentially lowers effective dosages. The current review examines the critical factors influencing oral PBs delivery, explores how biocompatible and biodegradable SLNs can be optimized to overcome these challenges, and discusses emerging techniques in nanoparticle design that could further enhance the efficacy of PBs delivery systems.

## Introduction and background

Phyto-bioactives (PBs) are naturally occurring substances in plants that exhibit significant biological activity and potential health benefits. These compounds are of immense scientific interest due to their diverse therapeutic properties, including anti-inflammatory, antioxidant, anticancer, and antimicrobial activities, which make them beneficial in promoting health and preventing disease. PBs show significant potential for developing new drugs and supplements to treat various ailments [1]. Dietary supplementation with PBs such as resveratrol, quercetin, curcumin, epigallocatechin gallate, catechin, and sulforaphane has consistently demonstrated health benefits. However, PBs face significant challenges in drug delivery and targeted effects due to poor stability, low solubility, rapid elimination, poor lipid solubility, and the need for validated isolation and purification processes. In herbal medicinal practices, the therapeutic effectiveness of drugs is often reduced due to limited target site delivery. The majority of the administered drug is distributed throughout the body based on its physicochemical properties. This widespread distribution results in a lower concentration of the drug at the desired target site, thereby reducing its therapeutic potency and efficacy. Consequently, achieving the intended therapeutic outcomes becomes more challenging [2].

Targeted delivery of herbal formulations remains a considerable challenge for many medicinal plants. For example, while tannins, flavonoids, and terpenoids are water-soluble, their ability to penetrate biological membranes is limited, resulting in poor absorption. Furthermore, their larger molecular size contributes to decreased bioavailability and efficacy. Addressing these challenges requires innovative delivery systems that can enhance the stability, solubility, and targeted delivery of PBs to improve their therapeutic effectiveness. To address these challenges, innovative drug delivery systems (DDS) have been developed for phytomedicines. These advanced DDSs offer multiple advantages, including site-specific delivery, improved solubility and stability, enhanced bioavailability, minimized toxicity, and controlled release profiles of bioactive compounds. Among these advanced DDSs, solid lipid nanoparticles (SLNs) have shown significant promise. SLNs can encapsulate PBs, enhancing their stability, solubility, and bioavailability while protecting them from rapid elimination and degradation. By using SLNs, it is possible to achieve more effective and targeted delivery, ensuring a higher concentration of active constituents reaches the desired site of action, thus maximizing therapeutic efficacy and minimizing systemic dispersion and potential toxicity [3].

SLNs are lipid-based delivery systems that vary in size, typically ranging from 30 to 1000 nm. They are formulated using readily degradable lipids and offer numerous advantages over other nano-delivery systems. SLNs ranging from 120-200 nm can effectively evade filtration by the reticuloendothelial system, particularly in the spleen and liver. This characteristic enables prolonged circulation and reduces the potential for both acute and chronic toxicity associated with rapid clearance or accumulation in these organs. They enhance bioavailability and productivity, ensure greater reproducibility, minimize the use of organic solvents in preparation, and offer enhanced stability for labile phyto-compounds or drugs. Additionally, SLNs have the capability to encapsulate both hydrophilic and hydrophobic compounds effectively. Furthermore, SLNs are formulated using bio-degradable lipids, ensuring biological safety, facilitating scalable production, enabling simple sterilization, and allowing prolonged storage periods. These attributes underscore the suitability of SLNs for oral delivery of several PBs, presenting a promising strategy to improve their therapeutic application [4].

The essential components of SLNs include lipids, which form a solid matrix at room temperature and encompass triglycerides, fatty acids, waxes, and steroids; emulsifiers used either alone or in combination to stabilize the nanoparticle dispersion and prevent aggregation; active pharmaceutical ingredients (APIs), which encapsulate drugs or therapeutic compounds; and a solvent system, typically an aqueous medium, for dispersing the nanoparticles. The selection and properties of these constituents critically influence the characteristics and effectiveness of SLN formulations, impacting their stability, bioavailability, and targeted drug delivery capabilities.

SLNs have revolutionized drug delivery by amalgamating the advantageous characteristics of polymeric nanoparticles, liposomes, and microemulsions. They are employed for various routes of administration, including parenteral, oral, ocular, and topical delivery, as well as for protein and peptide delivery. This review critically analyses the primary parameters and variables influencing the oral delivery of PBs and addresses challenges such as gastrointestinal stability and absorption efficiency. Furthermore, the review explores how biocompatible and bio-degradable SLNs can play a pivotal role in enhancing the chemical stability, aqueous solubility, and overall bioavailability of PBs. Additionally, it examines the potential of SLNs to enhance the specificity and selectivity of PBs, ensuring more precise therapeutic outcomes [5].

## Review

Challenges in oral absorption of phyto-bioactives

The oral administration of PBs faces several significant challenges that limit their therapeutic efficacy and widespread application. These challenges stem from the inherent physicochemical properties of PBs and the complex physiological barriers of the human digestive system. Primary challenges include:

Low Aqueous Solubility

Many PBs are hydrophobic in nature, resulting in poor water solubility. This characteristic severely limits their dissolution in gastrointestinal fluids, which is a prerequisite for absorption. Examples include curcumin from turmeric, resveratrol from grapes, and quercetin found in various fruits and vegetables. Their low solubility not only reduces bioavailability but also makes it difficult to formulate them into oral dosage forms.

Poor Biostability

PBs are often susceptible to degradation in the harsh gastrointestinal environment. Factors such as pH variations, enzymatic activity, and the presence of other food components can lead to chemical modifications or breakdown of these compounds before they reach their site of absorption. For instance, anthocyanins from berries are, particularly, sensitive to pH changes, while certain polyphenols can be degraded by intestinal microbiota.

Limited Bioavailability

The combination of low solubility and poor stability contributes to the limited bioavailability of PBs. Additionally, many of these compounds are subject to extensive first-pass metabolism in the liver, further reducing the amount that reaches systemic circulation. Some PBs also have poor permeability across intestinal membranes or are substrates for efflux transporters, which actively pump them back into the intestinal lumen.

Lack of Target Specificity

Once absorbed, PBs often distribute widely throughout the body rather than concentrating at specific target sites. This non-specific distribution can lead to reduced efficacy at the intended site of action and potential side effects in other tissues. For example, while resveratrol has shown promise in cancer treatment, its broad distribution in the body limits its concentration at tumor sites.

High Concentration Requirements

Due to the aforementioned challenges, achieving therapeutic effects with PBs often requires high oral doses. This necessity for high concentrations can lead to several issues like potential toxicity and formulation difficulties [6].

Solid lipid nanoparticles as carriers

**Figure 1:**
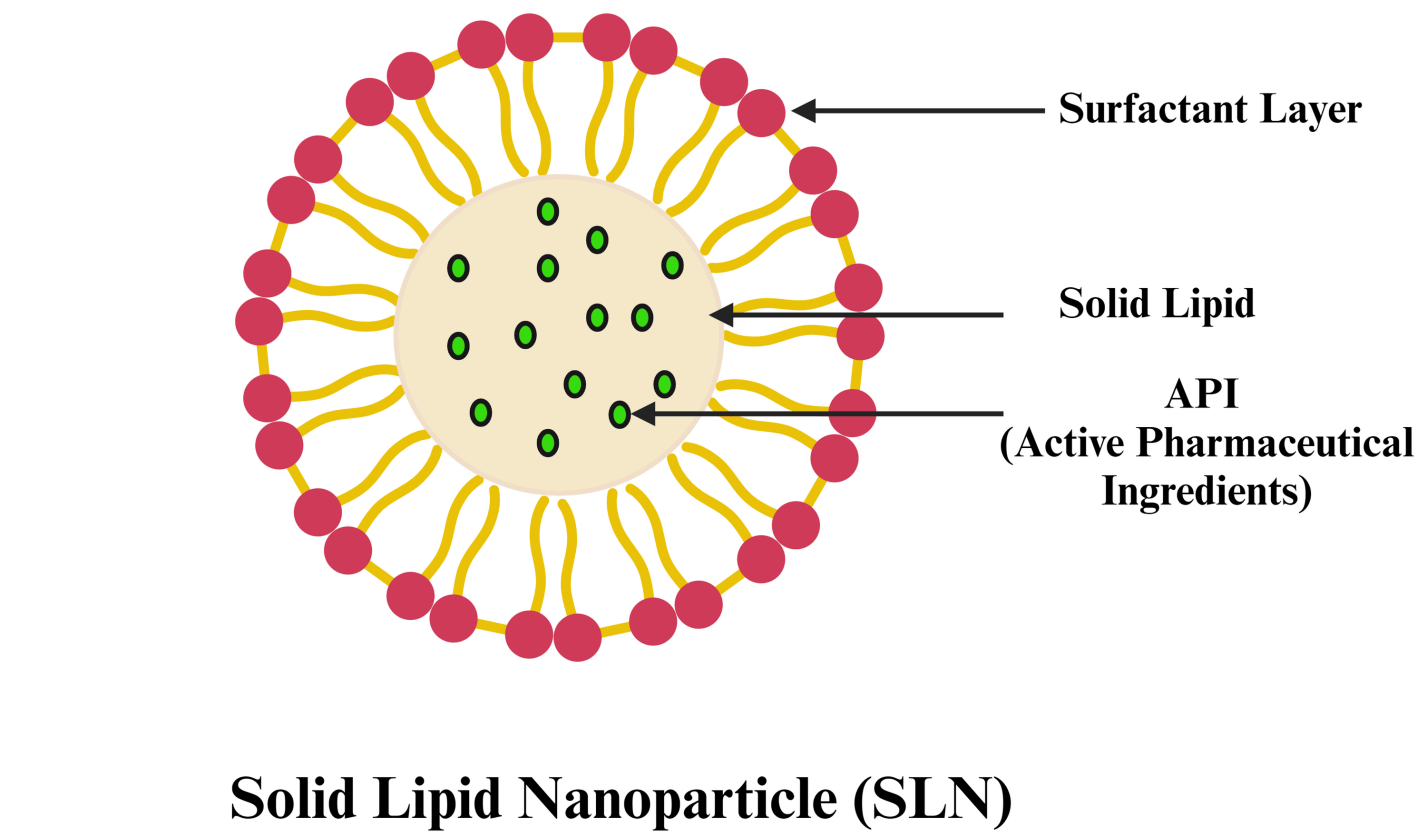
A schematic overview of the structure of SLNs SLNs: solid lipid nanoparticles This figure was created by the author (Gokul V).

Phospholipids are essential components of lipid nanoparticles as a result of their amphiphilic properties and biocompatibility. However, traditional carriers such as lipospheres, liposomes, and microsimulation exhibit notable limitations, including complex manufacturing processes, suboptimal encapsulation efficiency, and scalability issues. As a result, SLNs shown in Figure 1 have emerged as a more effective solution and are gaining significant attention as innovative colloidal drug carriers for various drug delivery applications. These submicron colloidal carriers, comprising physiological lipids dispersed in an aqueous medium or surfactant solution, demonstrate considerable potential for achieving targeted and controlled drug delivery, thereby addressing many of the shortcomings associated with traditional lipid-based systems.

**Figure 2:**
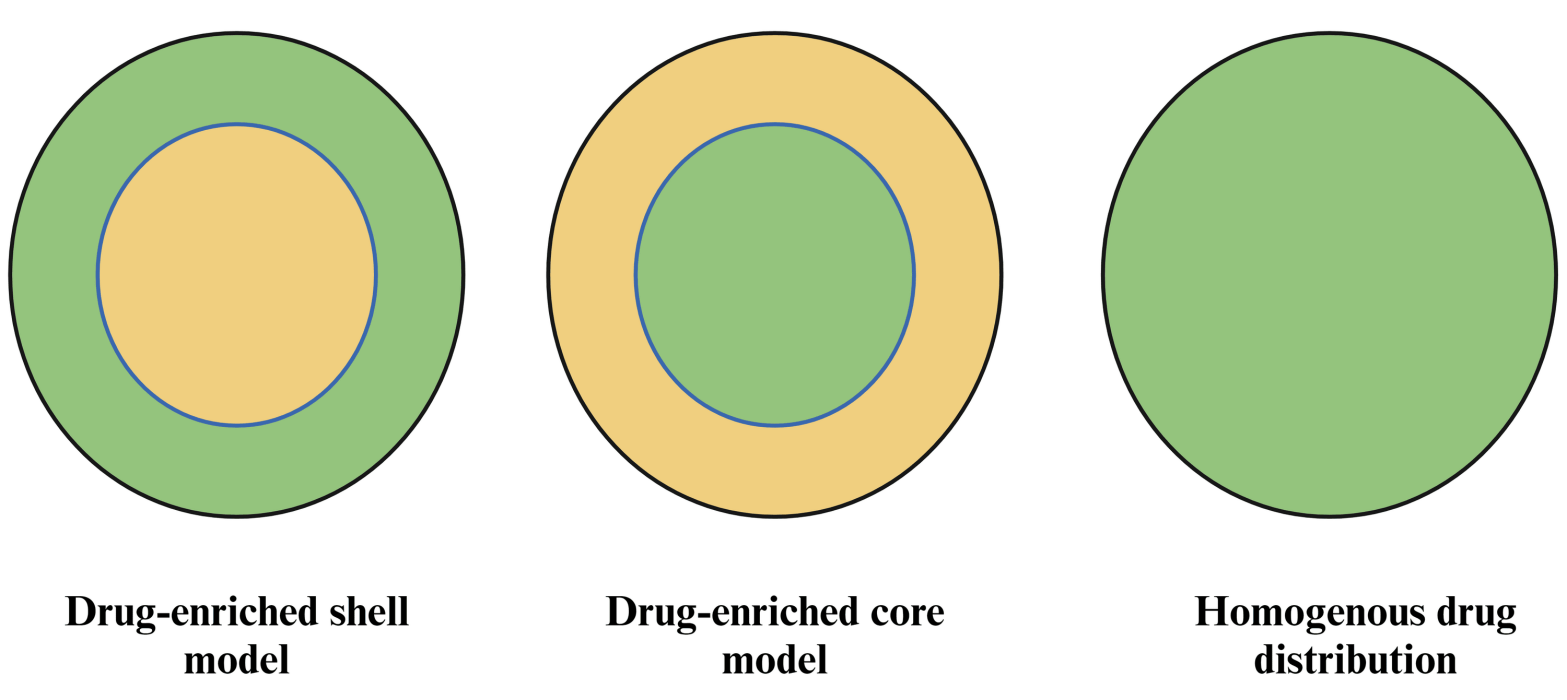
Drug incorporation models of SLNs SLNs: solid lipid nanoparticles This figure was created by the author (Gokul V).

Drug incorporation models in SLNs reveal three distinct morphologies based on the location of the incorporated drug molecules (Figure 2). The first model is the drug-enriched shell model, where the drug molecules are primarily located in the outer shell of the nanoparticle. The second is the drug-enriched core model, where the drug is predominantly concentrated in the core of the nanoparticle. The third model is the homogeneous matrix model, in which the drug is uniformly distributed throughout the entire matrix of the SLN. Each model impacts the drug release and delivery characteristics of the SLNs differently.

Formulation of solid lipid nanoparticles

SLNs are primarily made from lipids that serve as matrix materials, supplemented by emulsifiers, co-emulsifiers, surfactants, and water. Charge modifiers are added to enhance stability and targeting, thereby improving circulation time and precision in targeting specific areas. Below is a list of various excipients utilized in the development of SLNs.

Lipids

The core of lipid-based drug delivery systems (LBDDS) comprises various lipids, including fatty acids, acylglycerols, and waxes, either individually or in combination. This core is surrounded by a stabilizing layer of surfactants such as cholesterol, bile salts, and phospholipids. As the primary constituents of lipid-based nanoparticles, lipids play an essential role in determining drug loading capacity, stability, and sustained release behavior. Researchers have investigated lipid nanoparticle dispersions using various lipid materials, including mono- di-, and triglycerides, fatty acids, waxes, and steroids, which are generally recognized as safe (GRAS) and well-tolerated physiologically. Table 1 shows the list of various solid lipids used in the formulation of SLNs [7]. Selecting appropriate lipids is a critical step in the formulation of lipid nanoparticle dispersions. Although definitive guidelines are not established, practical considerations have been proposed as selection criteria. A key factor is the drug's solubility in the lipid, which serves as an empirical measure to guide the selection process. Drugs having higher solubility or partition coefficient in the lipids exhibit maximum drug load. Notably, drugs exhibit varying solubilities in different lipid matrices, resulting in diverse apparent partition coefficients. As a consequence, the same drug may achieve different loading capacities across various lipid matrices.

**Table 1:** Various classes of lipids used in the formulation of lipid nanoparticles

Class of lipids	Lipids
Fatty acids	Behenic acid, stearic acid, palmitic acid, and dodecanoic acid.
Triglycerides	Glyceryl tristearate, glyceryl trilaurate, glyceryl trimyristate, glyceryl tripalmitate, glyceryl tribehenate, and caprate triglyceride
Monoglycerides	Glyceryl monostearate and glyceryl hydroxystearate
Diglycerides	Glyceryl dibehenate and glyceryl palmitostearate
Waxes	Beeswax, cetyl palmitate, and solid paraffin
Sterols	Cholesterol
Glyceryl esters	Glyceryl behenate, glyceryl monostearate, and glyceryl trimyristate
Alcohols	Cetyl alcohol and softisan 142
Mixtures and others	Hardened fat (Witepsol E 85), monostearate monocitrate and glycerol, propylene glycol palmitic stearate, and softisan 142

A primary challenge with SLNs is their restricted ability to incorporate hydrophilic drugs, largely due to partitioning effects during the manufacturing process. Effective integration into the solid lipid matrix is generally achievable only for highly potent hydrophilic drugs administered in low doses [8].

Surfactants

**Figure 3:**
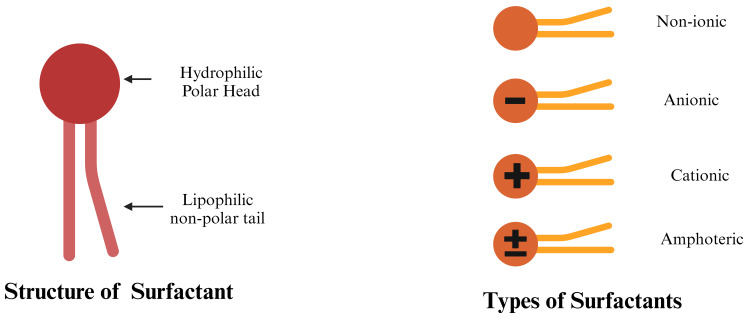
Diagrammatic representation of structure and types of surfactants used in the preparation of SLNs SLNs: solid lipid nanoparticles This figure was created by the author (Gokul V).

Surfactants are one more essential component in SLN formulations, playing a crucial role in enhancing colloidal stability during production. These amphipathic molecules consist of a hydrophilic head (polar) and a lipophilic tail (non-polar). This unique structure allows surfactants to adsorb onto surfaces or interfaces even at low concentrations. Surfactants are broadly classified into three main categories according to their charge; amphoteric, non-ionic, and ionic. Figure 3 shows the structure and types of surfactants.

In SLN manufacturing, surfactants help stabilize the particles, preventing aggregation and maintaining the integrity of the nanoparticle dispersion throughout the production process and beyond. The physical and chemical properties of SLNs are influenced by the composition and concentration of surfactants. Surfactants play two essential roles: they disperse the lipid melt in the aqueous phase during production and stabilize the SLNs in dispersions after cooling. Table 2 presents examples of surfactants from various classes used in the preparation and stabilization of lipid-based nanoparticles.

**Table 2:** Surfactants commonly used in the formulation of SLNs CTAB: cetyltrimethylammonium bromide; SLNs: solid lipid nanoparticles

Types	Surfactants
Ionic surfactants	Sodium dodecyl sulphate, sodium cholate, sodium oleate CTAB, and sodium glycocholate
Non-ionic surfactants	Polysorbates, sorbitan esters, poloxamer, and solutol HS15
Amphoteric surfactants	Egg phosphatidylcholine, soy phosphatidylcholine, egg phospholipid, and soy phospholipid

Co-surfactants

An ideal co-surfactant for SLNs is amphiphilic molecules that possess both substantial hydrophobic regions and high water solubility. This unique combination of properties allows these co-surfactants to effectively stabilize interfaces by maintaining a readily available pool of molecules at the lipid-water boundary, enhancing the overall stability of the nanoparticle system. Co-surfactants are commonly used with primary surfactants to enhance stability and control the properties of SLNs like polyethylene glycol (PEG) derivatives (e.g., PEG-40 hydrogenated castor oil) [9].

Emulsifiers and Co-emulsifiers

Emulsifiers are vital in formulating high-quality SLNs, as their concentration helps reduce surface tension and promote particle partitioning during homogenization, resulting in decreased particle size and increased exposed surface area. Ideal emulsifiers for SLN preparation should be non-toxic, compatible with other ingredients, able to achieve desired particle size with minimal quantity, and capable of ensuring SLN stability through surface coating. Common emulsifiers include polysorbates, lecithin, polyvinyl alcohol, sodium dodecyl sulphate, and pluronics, which can also enhance SLN circulation by blocking the reticulo-endothelial system and improving the delivery of drugs to the brain. However, emulsifiers alone may not suffice for maintaining SLN stability. During solid lipid recrystallization, phospholipid molecules in vesicles have limited mobility, potentially leading to particle aggregation and size increase. To address this issue, co-emulsifiers are employed to further stabilize SLNs and improve their properties. These can be ionic or non-ionic substances, such as glycocholate, taurocholate sodium salt, tyloxapol, taurodeoxycholic acid sodium salt, sodium dodecyl sulphate, sodium oleate, sodium glycocholate, cholesteryl hemi succinate, and butanol [10].

Cryoprotectants

Cryoprotectants are essential additives in the process of lyophilization, preventing the aggregation of solutes or suspended materials during freeze-drying. These substances include various sugars (trehalose, glucose, mannose, maltose, and lactose), sugar alcohols, amino acids, and polymers (PVP, PVA). Cryoprotectants form a protective matrix around active ingredients, preserving the integrity and stability of the formulation throughout the lyophilization process, thus ensuring product quality and efficacy [11].

Release of Therapeutic Agents From Solid Lipid Nanoparticles

The release of drugs from SLNs is governed by complex principles involving diffusion, erosion, and degradation of the lipid matrix. The release kinetics are influenced by the drug's physicochemical properties, its distribution within the SLN, and the lipid carrier's characteristics. Research has demonstrated that drug release profiles from SLNs are typically biphasic, with an initial burst release followed by a sustained release phase. The production temperature significantly affects this pattern, with hot homogenization at high temperatures typically resulting in a more pronounced burst release, while cold homogenization minimizes this effect. Surfactant concentration also plays a crucial role, with higher concentrations leading to increased burst release. This phenomenon is due to the redistribution effects of the active compound between the lipid and water phases during the heating and cooling stages of the hot homogenization process. Other factors influencing release include particle size, surface properties, lipid crystallinity, melting point, and the physiological environment of administration. Understanding these principles and effects is essential for optimizing SLN formulations to achieve desired release characteristics for specific therapeutic applications[12].

Fabrication of solid lipid nanoparticles 

SLNs are prepared using various techniques, each with its advantages and specific applications.

High-Pressure Homogenization

It is among the most commonly employed techniques for preparing SLNs. It involves the application of high pressure to push the lipid and aqueous phases through a narrow gap, resulting in the formation of nanoparticles.

Hot homogenization: The non-aqueous layer (lipid) is melted, and the drug is dissolved in it. This mixture is then homogenized at high temperatures above the melting point of the lipid. The process is efficient but can lead to drug degradation if the drug is heat-sensitive.

Cold homogenization: The drug-loaded lipid is first solidified, ground, and then homogenized at or below room temperature to avoid drug degradation. This method is suitable for thermolabile drugs and helps maintain their stability [13].

Ultrasonication

Ultrasonication utilizes ultrasonic waves to create cavitation forces that break down the lipid particles into nanoparticles.

Probe ultrasonication: Involves the direct application of ultrasonic energy using a probe, leading to effective particle size reduction. It provides high energy input and results in smaller particle sizes.

Bath ultrasonication: Uses an ultrasonic bath, providing a less intensive but more uniform energy distribution. This method is less aggressive compared to probe ultrasonication and is suitable for less rigorous particle size reduction requirements [14].

Solvent Evaporation

This method involves dissolving the lipid both lipid and active compound in an organic solvent, followed by emulsification within an aqueous medium. Subsequently, the organic solvent is removed through evaporation under vacuum conditions, resulting in the formation of SLNs.

Single Emulsion

The lipid-solvent mixture is emulsified directly into the aqueous phase, followed by solvent evaporation. It is a straightforward method but may result in lower encapsulation efficiency for hydrophilic drugs.

Double Emulsion

A water-in-oil-in-water (W/O/W) emulsion is formed, particularly useful for incorporating hydrophilic drugs. This technique enhances the encapsulation efficiency of hydrophilic drugs by entrapping them within the internal aqueous phase [15].

Microemulsion-Based Techniques

This technique involves the formation of a microemulsion which is then dispersed in cold water to precipitate the nanoparticles.

Formation: The microemulsion consists of a lipid phase, surfactant, co-surfactant, and an aqueous phase, which upon mixing, forms a transparent microemulsion. The process is carried out at a specific temperature to maintain the microemulsion state.

Precipitation: Rapid cooling of the microemulsion leads to the solidification of the lipid and the formation of SLNs. This method allows for controlled particle size and high drug-loading efficiency.

Double emulsion method: This method is especially suitable for encapsulating hydrophilic drugs. It involves creating a double emulsion (W/O/W), where the drug is initially dissolved in the inner aqueous phase.

Emulsification: The primary water-in-oil (W/O) emulsion is prepared by emulsifying the drug-loaded aqueous phase into the lipid phase. This primary emulsion is then emulsified into an external aqueous phase to form the double emulsion.

Solvent Removal

The solvent is removed, typically by evaporation, leading to the formation of SLNs. This method is effective in encapsulating hydrophilic drugs and provides a sustained release profile [16].

Several other novel techniques have been developed to improve SLN preparation and drug delivery efficiency. They are mentioned below:

Spray Drying

A rapid and scalable method where the lipid-drug mixture is sprayed into a heated chamber, evaporating the solvent and forming nanoparticles. It is appropriate for large-scale manufacturing and offers good control over particle size.

Supercritical Fluid Extraction

It uses supercritical CO_2_ to extract the solvent from the lipid-drug mixture, forming nanoparticles with minimal solvent residues. This method is environmentally friendly and provides high-purity nanoparticles.

Electrospray

It involves the application of an electric field to create a fine spray of the lipid-drug mixture, leading to the formation of SLNs upon solvent evaporation. This methodology enables fine-tuning of nanoparticle dimensions and structural characteristics [17].

Characterization of solid lipid nanoparticles

Characterization of SLNs is crucial to understanding their physical and chemical properties, which directly impact their performance in drug delivery. Several techniques and instruments are employed for the comprehensive characterization of SLNs, as discussed below:

Particle Size and Size Distribution

Dynamic light scattering (DLS): Measures the size distribution and polydispersity index of nanoparticles by analyzing the scattering of light by particles in suspension.

Laser diffraction: Determines particle size distribution by measuring the angular variation in the intensity of laser light scattered by particles.

Electrophoretic light scattering (ELS): Used to measure the zeta potential, which indicates the surface charge of SLNs and their stability in suspension. Higher zeta potential values (positive or negative) suggest better stability [18].

Morphology and Structure

Transmission electron microscopy (TEM): Provides detailed images of the internal structure and morphology of SLNs at the nanoscale.

Scanning electron microscopy (SEM): Offers surface images of SLNs, revealing their shape and surface characteristics.

Atomic force microscopy (AFM): Used to obtain three-dimensional surface topography and mechanical properties of SLNs.

Crystallinity and Polymorphism

X-ray diffraction (XRD): Analyses the crystalline structure of the lipid matrix in SLNs, indicating the degree of crystallinity and polymorphic transitions.

Differential scanning calorimetry (DSC): Measures the thermal properties, such as melting and recrystallization behavior, providing insights into the crystallinity and stability of lipids.

Drug Loading and Encapsulation Efficiency

High-performance liquid chromatography (HPLC): Quantifies the quantity of drug encapsulated within SLNs and determines the encapsulation efficiency [19].

Ultraviolet-visible spectroscopy (UV-Vis): Used to measure drug concentration in SLNs, particularly for drugs that absorb in the UV-visible range.

In Vitro Release Studies

Dialysis method: SLNs are placed in a dialysis bag, and the release medium is sampled over time to measure the drug release profile.

Franz diffusion cell: It is employed to investigate drug release through a synthetic or biological membrane, providing a better simulation of in vivo conditions.

Surface Properties

Fourier transform infrared spectroscopy (FTIR): Analyses the chemical interactions between the drug and lipid, providing information about the molecular structure and stability.

Contact angle measurement: Determines the wettability and hydrophilicity/hydrophobicity of SLN surfaces, which can influence drug release and interaction with biological membranes [20].

Stability and storage of solid lipid nanoparticles

The storage stability of SLNs can be assessed by monitoring various physical properties over time, including appearance, particle size, zeta potential, drug content, and viscosity. External factors, particularly temperature and light, play a critical role in determining long-term stability [21]. The zeta potential shall generally remain more than -60 mV to ensure physical stability, as significant deviations can indicate instability due to aggregation or changes in surface charge. Monitoring particle size over time helps detect aggregation or changes in nanoparticle distribution, which can compromise stability. Regularly measuring drug content ensures that SLNs maintain their therapeutic efficacy and do not undergo significant drug leakage or degradation. Visual inspection can reveal changes such as phase separation, precipitation, or color changes, indicating instability. Changes in viscosity can reflect alterations in the SLN dispersion, potentially indicating aggregation or degradation. Storage at 4°C is the most favorable, maintaining SLN stability and preventing significant changes in physical properties. Long-term storing at 20°C does not result in aggregation or drug loss, making it a viable option for stable formulations. However, storage at 50°C should be avoided due to rapid particle growth and significant instability. Understanding these factors and conditions helps in optimizing the shelf-life and efficacy of SLNs in drug delivery applications [22].

## Conclusions

SLNs have emerged as a promising strategy for improving the oral absorption of PBs. These naturally occurring plant-derived substances offer substantial health benefits, but their application is often hindered by low bioavailability, poor biostability, and limited aqueous solubility. The utilization of SLNs in DDS represents a significant advancement in the field, offering numerous advantages over traditional delivery methods. However, several challenges and opportunities for further research remain. Future perspectives should focus on improving SLN formulations to enhance drug loading capacity and stability, particularly for hydrophilic drugs. Advanced techniques in nanoparticle design and fabrication need to be explored to improve encapsulation efficiency and ensure consistent production quality. Furthermore, there is a need for extensive in-vivo studies to better understand the pharmacokinetics and biodistribution of PB-loaded SLNs, ensuring their safety and efficacy in clinical applications.

Additionally, investigating the interactions between SLNs and biological systems shall be essential for developing more effective and targeted delivery systems. This includes studying the impact of surface modifications and the use of targeting ligands to enhance specificity and selectivity. Advances in cryoprotectant technology and storage methods will also be essential to maintaining the stability and efficacy of SLNs over extended periods. Overall, while SLNs hold great promise for improving the oral delivery of PBs, ongoing research and development are necessary to fully realize their potential in therapeutic applications.
